# Narrative agency in retirement interviews: a qualitative longitudinal study

**DOI:** 10.1080/17482631.2025.2592419

**Published:** 2025-11-30

**Authors:** Hanna Kosonen, Pirjo Nikander, Kirsi Lumme-Sandt

**Affiliations:** a Faculty of Social Sciences, Tampere University and Gerontology Research Center, Tampere, Finland; b Doctoral School, Tampere University, Tampere, Finland

**Keywords:** Retirement, narrative agency, sense of the possible, older workers, qualitative longitudinal research

## Abstract

**Purpose:**

The outspoken aim of extending working lives faces the challenge of strong retirement age norms. This paper explores how older postal workers exercise their agency when narrating their retirement experiences and discusses implications for societal goals to extend working lives.

**Methods:**

Utilizing the tools of qualitative longitudinal analysis and narrative agency, we analyzed thematic interviews with three representative cases from a population of 20 former Finnish postal workers.

**Results:**

The three cases demonstrated narrative agency by A) questioning dominant social norms regarding unemployment and personal value, B) grappling with unexpected changes to life in retirement, and C) narrating changes to work and one’s own focus in life, paired with a freedom to choose one’s activities.

**Conclusion:**

Updating negative dominant societal narratives about aging and work is pertinent in the endeavor to extend working lives. Retirement is a “space of the possible.” Developing workplaces into such spaces through increased structural flexibility could improve the image of aging at work.

## Introduction

Extending work-lives, working beyond the current retirement age, is a widely recognized concern at the OECD, EU and state government levels in the rapidly ageing affluent “West” (OECD 2006; Eurofound 2019; Egdell et al., 2020). Despite favorable legislation, research has frequently observed a lack of age management practices, such as retainment, retraining, or recruitment, to support longer work lives (Egdell et al. 2020). Further, research shows that older workers and their employers alike may have negative beliefs about older workers (Pärnänen, 2012; Vickerstaff & Van der Horst, 2021) and this limits older workers’ employment opportunities. Employment officials may also demonstrate a lack of care and an indifference for the re-employment prospects of people nearing retirement age (Keskinen, 2024, p. 84; Bowman et al., 2016; Laliberte Rudman & Aldrich, 2021). Retirement is popularly conceptualized as a source and time of freedom (see e.g., Apouey, 2022; Grødem & Kitterød, 2022; Repetti & Calasanti, 2018).

Flexibility appears in the literature as a recommended measure for sustainably extending work lives (Allen et al., 2021; Loretto & Vickerstaff, 2015). Allen et al. (2021) found that “FWAs [flexible work arrangements] may be effective in reducing the perception and impact of negative stereotypes” on work engagement (p. 413). Further, extending flexible practices beyond employees of a particular age could reduce ageist perceptions regarding HR policies specifically targeting older employee needs (Allen et al., 2021) and make disclosing any need for and receiving support measures easier (Gignac et al., 2022). Flexibility and adjustment to people’s individual life situations and capabilities at work, regardless of age, could also serve younger employee needs (see Nilsson, 2020). Creating a more sustainable work culture is important, considering the increasing trends in European mental health-related disability pensions reported in the past two decades (ETK 2020; Gustafsson et al., 2014; Karolaakso et al., 2020, Prins, 2006).

Motivated by Higgs et al. (2003), who described the marketization of the British civil service in the 1980s in ways that parallel changes to Finnish public services from the 1990s through the early 2000s (Kantola, 2006) and beyond, this study focuses on interview data from retiring Finnish postal service workers. Research has also identified large-scale structural changes driven by a neoliberal logic in Finnish work life (Gould & Saurama, 2003; Kantola, 2006). These include a paradigm shift in public discourse and policy regarding work life participation at older ages from a “right to retire” to a “duty to work,” between the 1980s and 2000s (Gould & Saurama, 2003). The Finnish postal services are a state-owned organization and a useful example, even a microcosm, of neoliberal changes to the Finnish welfare state (Gould & Saurama, 2003; Kosonen & Keskinen, 2019). Similar neoliberal political climates have been identified in e.g., UK and Germany (Hofäcker et al., 2016).

Efforts to extend work-lives could take stock of the aging concepts used to negotiate delayed (or, for that matter, early) retirement. This is supported by a recent research project conducted among 61-year-old Finns (*N* = 206), representing the Finnish birth cohort of 1959. Findings show that only approximately 20 per cent of the participants were retired (Kokko et al., 2024), 70 per cent considered their health to be either very good or fairly good (Saajanaho et al., 2021), and 62 per cent had a subjective age lower than their chronological age (Kokko & Saajanaho, 2024).

Although many Finns in their early 60s report feeling that they are younger than their age, they still tend to retire in their early to mid-60s. From 2013 through 2023, the average Finnish retirement age rose, with some peaks and valleys on the way, from 60,3 to 61,1 years; median retirement age rose steadily from 63,1 to 64,3 years (ETK, 2024). The participants in the present study (born in 1950−1962) represent a Finnish birth cohort whose statutory retirement age is defined by birth year (e.g., for people born in 1959, the statutory retirement age is 64 years and three months (ETK, 2019)).

This study offers insight into the work exit process and transition into retirement by applying qualitative longitudinal research (QLR) to a population (*n* = 20) of Finnish postal workers leaving full-time employment. Using these data, the analyses directly resonate with trends and recent developments across Europe. Data collection entailed repeated interviews by two interviewers with the participants between 2015 through 2018, either face-to-face or on the phone. Each individual participant’s interviews were conducted in Finnish by one and the same researcher, to build rapport and thus enable participants to express themselves more freely.

The methodological focus is on narratives used around ageing at work and retirement discussions because narratives, in part, uphold and reproduce limiting structures in the form of preconceived notions and stereotypes (Laceulle, 2018; Tulle, 2004; Meretoja et al., 2022). This study explores narrative sense-making and agency as part of the work-exit process. Narrative agency could be a remedy for limiting narratives related to ageing, as it involves becoming aware of, questioning, and broadening existing narratives.

The methodology of this study is motivated by Neale's (2019) assertion that QLR is uniquely suited to understanding unfolding transitions and the ways in which “…individuals overwrite their biographies, continually adjusting the narratives of their lives to their evolving experiences” (p. 119). The QLR research design, paired with narrative analysis, holds great potential for better understanding the role of narratives during change, such as retirement transitions.

Studying stories told in society about the role of work and retirement in a person’s life can illuminate the challenges societies face in extending their work-lives. One of the aims of this study is to better understand older workers agency in the ways that they narrate their work exit and retirement processes in retrospect. Another is to see if this could be studied through a model of narrative agency originally intended for analyzing works of fiction (Meretoja et al., 2022). Combining a narrative take with a longitudinal perspective, the analyses allow teasing out nuances in participants’ sense making practice and changes therein over time.

### Theoretical background

One of the enduring philosophical ideas in narrative research is that the stories people tell about themselves also shape their lives. Andersen et al. (2020) point out, however, that “relatively little research has explored the relationship empirically” (ibid, p. 368). The present study contributes to breaching this gap and claims that one of the central ways in which people make sense of changes, the world, and their lives is through narratives (Laceulle, 2018). People make decisions by reflecting on what is possible, and this is partly delineated by the “narrative environments” in which they live (Meretoja et al., 2022). Tulle (2004) and Laceulle (2018) note that narratives play a key role in the agency of aging individuals and emphasize the importance of questioning harmful and limiting narratives about aging. For these reasons, it is useful to examine narrative sense-making at the hinge points of a person’s later life, such as retirement, in relation to the structural, surrounding forces that underpin such events.

The master narratives present in each society perpetuate themselves through “an individual’s experiences,” which they also direct (Niemi & Komp-Leukkunen, 2022). One such master narrative observed among Finnish older workers is that “simultaneous changes at the workplace and in their health lead older workers to look for ways to exit their jobs’ (ibid., p. 1165). Master narratives, by nature, gloss over individual variation and detail. (ibid.). For this reason, a non-subsumptive way of studying narratives, such as the narrative agency model detailed by Meretoja et al. (2022), offers ways to pluralize and widen the image of ageing and agency. Doing so entails questioning the limiting model stories of retirement and widening the opportunities open to people at this transition point.

The theoretical framework of this study adopts Meretoja and colleagues’ (2022) narrative agency as its starting point. Of particular interest here is their concept of the “sense of the possible”, that is, a “sense of how things could be otherwise” (ibid., p. 393). Meretoja et al. (2022) direct researchers to look for what participants see as possible (or inevitable) for themselves and how they negotiate their position in relation to it. Table I presents the “central dimensions” of Meretoja and colleagues’ (2022) narrative agency model, followed by a commentary on how this relates to the study context.

**Table I.** The components of narrative agency by Meretoja et al. (2022, p. 392−393)

Narrative awareness	An “awareness of culturally available narratives that shape people’s lives by functioning as models of sense-making”, including *self-understanding* and *perspective awareness.*
Narrative imagination	Meaning “our ability to imagine beyond what appears to be self-evident in the present, to creatively and critically engage with cultural narrative models, and to imagine different narrative trajectories for oneself, one’s community, humankind, and the planet more broadly”, including “our ability to cultivate our *sense of the possible”* and 'to engage in *explorative ethical inquiry (…)*about basic existential issues (e.g., of what good life might mean), issues that lack definitive answers but are crucial to how we orient ourselves in the world’ (emphasis added).
Narrative dialogicality	Meaning “the narratively mediated process of how we become who we are in relation to other agents in the world—in a fundamentally dialogical and relational way”.

In the context of Finnish postal services in the 2010s, “culturally available narratives” include potential institutional narratives about what is typical, possible, or has already happened during a process called “collective negotiations’ (see Jolkkonen et al., 2012), which led to massive layoffs and personnel reductions in the participants’ organization (see Kosonen et al., 2020). Individual agency is always ultimately bounded by institutional “musts” (structural limitations) and “internal musts,” i.e., values internalized during socialization (see Flaherty, 2012), regardless of one’s potentially taking an agentic view of the proceedings, in the moment or in retrospect.

### Data

The data consist of semi-structured, longitudinal interviews conducted between 2015 through 2018 among 50−65-year-old former postal workers (*N* = 40, average age: 58,4 years, on average 34,1 years worked in Posti, 4,5 interviews on average). All participants had recently left Posti because of collective negotiations (see Jolkkonen et al., 2012). The data collection took place as part of a larger research project conducted at Tampere University and focused on economic inequalities among retired Finns (the Towards Two-Speed Finland? (2tS) research project). Participants were recruited in collaboration with a gatekeeper from Finnish postal services (henceforth, *Posti*, the conveniently brief Finnish-language name of the organization. Participants who volunteered were interviewed at regular intervals throughout the study period (2015−2018) by two interviewers. Participants were also able to contact the researchers and request an interview or otherwise inform the interviewer of meaningful life events or changes.

Further, our focus in this analysis was on participants who no longer sought full-time re-employment but rather opted to retire after the collective negotiations at Posti. The total number of interviewees is 20 and the number of interviews 96 (3−11 interviews/participant, 4,5 interviews on average). The 20 participants’ age range was 54−64 years during the initial interviews (average age: 60,5 years). Out of these 20, three cases were selected for detailed analysis. The three were aged 59−62 years during the initial interviews, and the total number of interviews was 12. There was great variation between individuals in terms of life situation and orientation towards work life. In the context of this study, “older worker(s)” is shorthand for people aged 50 years and older (based on the 2tS research project data collection parameters) and not yet fully retired during the initial interviews.

For this study, we selected three representative participants based on the combination of circumstances and agentic orientations the participants demonstrated towards their work exits, i.e., whether they left Posti voluntarily or involuntarily and what kinds of push/pull factors were at play, Table II explicates the entire data selection (*n* = 20), showing that the three cases under scrutiny in this study are a part of a larger body of data. The selected cases corresponded to most features in their respective groups and together showcase the diversity of life situations and retirement circumstances present in the data (Neale, 2019, p. 55, informs the sampling strategy).

**Table II.** Work exit agency types and their sub-types (modified from Kosonen & Keskinen, 2019).

Work exit agency type	Aggregate description of participants’ experiences	Respondents (M/F + code and pseudonym), grouped by volitionality of work exit
A: Being cast aside	Perceived lack of appreciation from employer after decades of service. A sense that working until retirement would have been more honorable than being let go and/or unemployment before retirement. Resistance to aspects of the work exit process.	Involuntary dismissal: F16 Liisa, F24 Siru, F20 Hannele, F36 Ulla, F21 Kaisa
B: Impaired work ability + financial opportunity	Reduced work ability due to physical problems, severance package perceived as an opportunity for early work exit.	Voluntary resignation: F1 Mari, F8 Anna, F22 Maikki
C: Personal time to leave + financial opportunity	Retirement age was close and announcement of severance package for voluntary resignations served as final push. Added push from negative changes to work and constant co-operative negotiations.	Voluntary resignation: M30 Veli, F25 Helvi, M4 Pentti, F28 Maisa
D: Avoiding impending layoff by resigning + financial opportunity	Perceived retirement age norms and thinking one would be dismissed anyway; decision to leave on one’s own terms when presented with severance package to voluntary resignations. Added push from frustration or disillusionment with work.	Voluntary resignation: F25 Helvi, F34 Leena
E: Refusal of subpar job offer and taking it easy	Being offered a new work position but perceiving it as somehow subpar and declining; resulting resignation or dismissal with severance package. Impending organizational changes as a push factor. Preserving one’s health as an added push factor (M9, M13).	Voluntary dismissal: M9 Ismo, M11 Petri; Involuntary dismissal: M13 Pertti, M7 Jyrki Voluntary resignation: M6 Ilmari
F: The outliers	Managerial push to leave work because retirement age was close and work demands would be too much for someone his age (M2); estimates he would have retired soon anyway due to having numerous side-jobs (part-time) (M5).	Voluntary (but influenced) resignation: M2 Mika; Voluntary resignation: M5 Juho

Researchers who were otherwise not involved in the production of this paper conducted the interviews and a third-party company transcribed them verbatim. The interviews were typically 45−120 minutes in duration and centered on participants’ experiences of leaving Posti and transitioning into life in retirement. Initial and final interviews were conducted in person, and any interviews in between via telephone by two researchers not involved as authors in this study. The data excerpts are translations by the corresponding author without back-translation. The quality of the transcriptions and translations were further tested in joint data analysis between the authors to provide inter-researcher validity (see Nikander, 2008 for details on data translation). The corresponding author also conducted the initial analyses, and the co-authors provided valuable commentary and assisted in editing the paper. They also contributed significantly to the design of the larger research project that provided the data (2tS research project).

## Method

Having already familiarized ourselves with the data set (*N* = 20) during our previous study (Kosonen et al., 2020), and subsequently with Meretoja and colleagues’ (2022) model, we selected three participants whose respective combinations of circumstances around and attitudes towards their work exit situations made them suitable representatives of larger groups of participants in the data set (for a quick overview of the whole data set, please refer to Table II).

We then performed a further, closer reading, driven by Meretoja and colleagues’ (2022) model on these three selected participants’ longitudinal interviews. Our aim was to see if a) Meretoja and colleagues’ (2022) model could be applied to the analysis of interview transcripts (as opposed to works of fiction) and b) how the model might illuminate potential changes in older workers’ agency over time, and demonstrate the ways they narrate their work exit and retirement processes in retrospect.

Several interviews in the data presented common themes centered on the retirement process. In Henderson and colleagues’ (2012) case history method, “individual archives are transformed into a single analytic narrative (ibid., p. 2). Based on a thorough reading of the data, “themes and motifs emerging from the interviews,” and “a narrative for each interview” were identified, and case histories were formulated for each of the three selected participants (see Henderson et al., 2012). These are presented as narrative summaries at the beginning of each case under Analysis and Results. The narrative summaries serve as illustrative cases of the sense-making processes present in older postal workers’ reports of leaving work life behind, as well as the three work exit types identified in the data in our previous study (see Table II). We have also included examples from elsewhere in the data set to demonstrate the prevalence of certain themes across participants.

The qualitative longitudinal research (QLR) design that produced the data (2TS) is informed by McLeod and Thomson's (2009) notion of “walking along” the participants and capturing socially and temporally situated personal processes. By inviting participants to reflect on their past and possible futures, QLR captures the process through which the self is made and remade over time and how cultural and personal narratives intertwine. This enables researchers to straddle the line between the micro and macro levels of society, where agency is also located (Kosonen et al., 2020).

QLR makes a narrative approach exceptionally suited for the processual analysis of decision-making and agency (Neale, 2019). This study explores how participants make sense of their work exit and retirement processes through the exercise of narrative agency, particularly focusing on the “sense of the possible” that participants demonstrate narrating their ongoing retirement processes (Meretoja et al., 2022). This dimension of narrative agency ties back to the QLR’s ability to capture socially situated personal processes (McLeod & Thomson, 2009, p. 77−79). A “sense of the possible” (Meretoja et al., 2022) is necessarily delineated by social structures: possibility is a structure-bearing dimension of individual agency (Kosonen et al., 2020).

The methodology chosen is an excellent fit for the in-depth, longitudinal exploration of a smaller sample of cases that seeks to illuminate the more general without unduly absorbing the particular into it (Meretoja, 2017, p. 110). Indeed, Henderson et al., (2012, p. 2) posit that individual case histories can function as “a key to unlock the social.”

All participant names are pseudonyms, and all placenames, references to specific job titles, work units, and any other recognizable details are anonymized. According to the Finnish National Board on Research Integrity TENK guidelines (2019, p. 19), this study’s research design did not require a human sciences ethics committee review. Informed consent was obtained from the participants in writing and re-checked at different phases of the follow-up.

## Analysis and results

This study explores the following work exit types identified in a previous study (see Table II, based on Kosonen & Keskinen, 2019). These cases differ in the level of voluntary-involuntary exit from work force and therefore capture the key characteristics of potential responses in this transition.

Case A: Involuntary layoffs are cases in which participants have lost their jobs against their desired outcome from collective negotiations.

Case B: Voluntary resignations are cases in which the participant chose to accept a financial arrangement and resigned from their job, receiving a lump sum of six months’ wages as compensation.

Case C: Voluntary layoffs are cases in which the participant refused a job offer in what appears to have been an amicable understanding with the employer. The participant was then made redundant based on financial and production-related rationale and given a lump sum of up to one year’s wages as compensation.

Voluntary cases tended to involve both self-serving (enjoying life, leaving a dissatisfying job) and altruistic motives (vacating the job for someone else) in the retelling.

Tables III, IV, and V contain information regarding interview timing through the follow-up for Cases A, B, and C, respectively.

**Table III.** Interviews, F21 Kaisa.

1^st^ a little over one month after leaving work at Posti because of layoff.
2^nd^ almost three months after the first, soon after being granted a part-time disability pension, a little over four months since work ended.
3^rd^ a little over eight months after the second interview, a little over one year since work ended.
4^th^ at four months and two weeks after the 3^rd^ interview, a little over 17 months since work ended, with approx. one year and nine months remaining until retirement.

**Table IV.** Interviews, F34 Leena.

1^st^ at seven months and two weeks after work ended.
2^nd^ at five months after previous interview, just over one year since work ended, has been fully retired approx. two months.
3^rd^ at six months after previous interview, a little over 18 months since work ended, has been fully retired approx. eight months.

### Case A: F21, Kaisa: shame followed by acceptance

#### Narrative summary

Kaisa’s case is an example of someone who initially feels ashamed about being unemployed but gradually accepts it. In her first interview, Kaisa demonstrates an awareness of the “culturally available narratives that shape people’s lives by functioning as models of sense-making,” that is, narrative awareness (Meretoja et al., 2022, p. 329) related to her unemployment status following her layoff from Posti. Kaisa does not yet appear to know what to do with her time; throughout the follow-up, she exercises narrative agency regarding her daily life in that by the third interview, she had gradually moved away from a sense of shame regarding her status, even embracing the label of the unemployed person to an extent. In her fourth interview, she describes feeling guilty over not going out every day but also acknowledges that this guilt is her own; the implication, perhaps, is that she need not necessarily feel this way. She also discusses a perceived norm with which she does not wish to comply, effectively engaging in ethical inquiry, a feature of narrative imagination (Meretoja et al., 2022).

In her first interview, Kaisa reports on her feelings of shame over her unemployment status and on having applied for a part-time disability pension, because she thinks it is more honorable to be on a disability pension than unemployed.

A: …Now I have been applying [for jobs], I have an application, three and a half months have passed since I applied for the part-time disability–Now we’re applying for the part-time disability pension.

Q: Right.

A: If I got that then I’d be like I’m a person too since I’m on pension.

Q: Oh right.

A: And not unemployed. I think it’s shameful to be an unemployed job seeker.

Q: Where does that feeling come from, do you know?

A: Gosh, I’ve always been proud of it, and I remember even as a young girl since [my hometown in northern Finland] always had a very high unemployment rate, and I was proud in the sense that I’d always done my job so well that I [always] had work. That I didn’t have to. Unemployed people are always [maligned as] lazy, always laying on a couch at home, spitting up at the ceiling and getting money from the social welfare office. I’ve never had to be like that, I’ve always been allowed, I’ve been allowed to work.

(F21 Kaisa, Interview 1 (Q = interviewer, A = participant)).

The final two sentences (underlined) in the above excerpt contain both the dominant cultural narrative of unemployment as a personal source of shame and an implicit reference to its counternarrative: unemployment as a structural issue (see Eichhorst et al., 2018). Kaisa having been *‘allowed’*, i.e., having had the opportunity to work, is at least partly structural. That is, acknowledging the unfair labeling of the unemployed, she states that she herself has, until now, been able to work in the system and survive the labor market structures.

Indeed, in her next turn, Kaisa recognizes her thoughts on the matter as divided between a personal sense of having lost her value as a person, knowledge of the cultural narrative that devalues unemployed people, and her personal stance that all people are equal in value.

- Q: Yeah. So does it feel like if you’re categorized [into] a group of people you don’t feel like you’re [a part of]?
- A: I have even said that I am an unemployed loser. Meaning this lay-off. I felt like my value as a person somehow crumbled. Like, unemployed people are viewed as nothing. Except, I’ve always thought of all people as being equal. But I know how people feel about the unemployed. So, my thoughts on this topic are quite divided.
- (F21 Kaisa, Interview 1)

Kaisa recognizes the dominant cultural narrative and how it has an impact on her opinion of herself, even though she states that this is not how she thinks of unemployed people overall. To use Meretoja and colleagues’ (2022) terms, this negotiation appears to include both self-understanding and perspective awareness but does not entirely liberate Kaisa from shame. She appears to simultaneously recognize that someone in her position does not need to feel ashamed but still cannot escape it altogether.

The sense of shame was a recurrent feature across the data set. In the following, Helvi (F25) also articulates difficulty in adjusting to the idea of unemployment,

- I: If you think a little more about this unemployment, do you see any good sides to it?
- P: Well I don’t know what I’d call a good side… I mean it does at least make life more free. Then again I’ve always worked, so it feels somehow undeserved, the unemployment benefit, like why am I also being paid since…
- I: So there are these conflicting thoughts.
- P: Right, yeah, and I shouldn’t think that of course, like I have been at work and paid my share of taxes, I’m sure.
- (Helvi, F25, Interview 1)

In her second interview, Kaisa expresses anger and bitterness towards her former employer for not retaining her, and the HR department for approaching her with messages about open positions like her old job. She is offended by the very notion of applying for a job she was fired from but says she is actively trying to avoid becoming bitter. She is aware that her former employer is obligated to send these messages, but it still rankles her, and she resists them by not responding, taking the stance that she will apply for a job if she wants one.

Kaisa also speaks of a recent contact with the unemployment office and how, after 40 + years of working in Posti, she would not know where else to work. During this reported interaction, Kaisa explicitly asked the unemployment official *“You’re not going to offer me work, are you?”* adding that she would not be interested. Kaisa is aware of Posti constantly looking for workers and the likelihood of being offered something through the unemployment office but frames this as “getting her orders,” rather than as a pleasant opportunity.

In her third interview, Kaisa confirms that she now thinks of herself as unemployed, not retired, and reports no longer feeling ashamed to tell people she is unemployed when asked. However, there are some apparent caveats to this, as she also routinely tells people that the layoff was not her fault, nor her wish, and that she is also on a part-time disability pension and, thus, not a *'total unemployed loser.’*

Kaisa also narrates a chance encounter with a former manager and how it impacted her when she was pointedly not greeted, as they crossed paths.

- Q: So, unemployment does come with this idea of you being a loser in some way.
- A: Right, yes; it does feel a bit like that. There’s this added thing where, in the summer, I was on an evening walk with one of my grandchildren and one of my managers, [name], walked towards me and like there were not that many others beside us, her with her husband, turned her head the other way, when it was time to say hello. That’s when it felt again like goddamn, I must have been pretty shit, since she cannot even greet me anymore. But then I thought of it like, oh well, that’s her showing her own stupidity. I suppose, or, like civilized people, greet each other when they see anyone remotely familiar.
- (F21 Kaisa, Interview 3)

Her initial sense appears to have been perceived as a bad employee or person, pivoting to acknowledge how the manager’s behavior in this situation was quite rude. This is followed by a final return to how she herself could have been perceived as a “loser.”

Kaisa reports that she is not particularly happy to tell people about her unemployment status, but she will tell them that she is unemployed rather than retired when asked.

- Q: Are there any other situations in which you maybe felt the unemployment in some way?
- A: There’s always this, I think it’s difficult when you have to explain to people out there when they ask, are you on vacation, or at work. So, I’m not overjoyed to say it, even now. But I do tell them nowadays. Specifically, I tell them first that I’m unemployed. But I don’t, I don’t voluntarily talk about it. If no one asks, I don’t say it.
- (F21 Kaisa, Interview 3)

The narrative renegotiation over time in these reported interactions still echoes the conflict present in the first interview: a perception of public opinion on unemployed people and her own experience with the identity of the unemployed person. The shame appears to have somewhat diminished or become more manageable, but there is still a perceived necessity to remark on her being on a part-time disability pension when talking to people she meets. She, however, does not identify as a pensioner, part-time or otherwise, but as an unemployed person.

When asked about her thoughts regarding work, Kaisa explains that her re-employment prospects are limited by her language skills and work experience. She also has no intention of returning to Posti for work.

In her fourth and final interview, Kaisa reports having yet to figure out what to do with her new-found freedom and wonders why she gets a “guilty conscience” if she does “not get out of the house for a couple of days.” She confirmed the interviewer’s estimation of the role of norms in her situation and concludes that she is probably the only one to blame herself in this way.

- Q: That is very interesting, how your being is regulated after all by these, some types of norms. That is how that sounds to me, very much.
- A: Well yes, and like why do you feel so bad if you don’t go anywhere for a couple of days, a terrible racket sounding from the conscience up there like, goddamn, you had 24 hours and what have you done, nothing [laughs], and the biggest thing making you feel guilty is your own self, like I’ve been thinking about that too, how I’m trying my hardest to come up with things to do, should I go to the gym although I hate it, shall I start going to some crafts club although I hate it, like do I really have to. Like, why couldn’t I [just] be. Like would I be a better person if I went to the gym or some volunteer job, probably. Supposedly, like a better person, so you wouldn’t berate yourself all the time. I don’t think anyone berates me but myself [laughs].
- (F21 Kaisa, Interview 4)

This is yet another example of Kaisa recognizing (but still somewhat falling victim to the dominant cultural norm regarding personal value and productivity present in her first interview) that her guilt is probably her perception alone. In Meretoja and colleagues’ (2022) terms, Kaisa exercises narrative awareness. She questions having to do something against her will and the pervasive social norm to stay active (see Foster and Walker (2015) on an overview and critique of active ageing). Refusing to be “a better person” in prescribed ways she personally dislikes, Kaisa engages in what Meretoja et al. (2022) term “explorative ethical inquiry,” i.e., grappling with complicated questions that impact people’s way of being “in the world,” such as “what good life might mean’ (Meretoja et al., 2022, p. 393).

### Case B, F34, Leena: resigning voluntarily but failing a leisurely descent into retirement

#### Narrative summary

In Leena’s case, the expectations and reality of her retirement clashed quite heavily. In her first interview, Leena reports deciding to pursue a life she would prefer to live by vacating her job. This also involved consideration given to her younger colleagues. Ultimately, however, Leena’s agency was structurally bounded as collective negotiations and employment regulations combined to provide an impetus for her to leave when she did. Furthermore, her work motivation had already declined due to changes in work duties. This points to structural changes, as does her perception of how the employer treated the employees.

By her third interview, Leena articulates what might be termed as an unfulfilled “sense of the possible” (Meretoja et al., 2022), the leisurely descent into life in retirement she had envisioned for herself. This plan was impeded by her husband’s sick leave and her needing to attend to the related paperwork. She describes this as a “crisis” which could be indicative of a culturally dominant “retirement as freedom” narrative (see e.g., Apouey, 2022; Grødem & Kitterød, 2022; Repetti & Calasanti, 2018), also present elsewhere in the data. She took initiative, however, by creating opportunities for herself to “break away” from “being programmed” by another: taking up regular exercise classes.

In her first interview, Leena summarizes her thinking as deciding to step down from work to avoid unnecessarily prolonging an unpleasant situation. She also reports considering herself already on part-time pension, adding her consideration for her younger coworkers who might need the job more than she does (see Vickerstaff & Van der Horst, 2021).

- Q: How did it go in your case, leaving [Posti]?
- A: We had collective negotiations, and they offered this [financial arrangement] program, which also included the possibility of taking the six months’ wages. I concluded that I have such a short time left until retirement, like what am I dragging on here for. Before that, I was already on a part-time pension, and I worked three days a week. Then, I thought, since these negotiations were about our unit, I started thinking that there [are] younger [people here] and for them it’s more important to keep the job.
- (F34 Leena, Interview 1)

Here, Leena combined a decision made in self-interest with an additional, more altruistic motive. Similar motives related to intergenerational solidarity can be found elsewhere in the data, for instance the interviews of Mari (F1) and Juho (M5), among others:

And there are no jobs here these days, even for young people or educated people (…) and we were able…actually I made the decision, it was influenced by this, when I decided to take the [severance package], (…) I imagined, if we’re being honest, I childishly though [this] and maybe there was something to it. I knew the [situation] so well and I knew my own circumstances (…) and my own likelihood of making it through, that (…) even if the wage was not all that great, but I knew that for a lot of people it would matter more than to me and they would not have survived without it. And I imagined that since there is a set amount of work that [needs to get done], that there would be enough of it for these younger people, so they [wouldn’t] put them out on unemployment (…).
(Mari F1, Interview 1)
(…) I don’t think the retirement age should be raised because then young people will never get a chance to work. That there’s exactly the thing that, I would look at the length of a person’s work career and that would be the decider. For when you retire.
(Juho M5, Interview 1)

Part of Leena’s motivation to apply for the voluntary resignation program was the risk of otherwise being laid off and ending up with a duty to accept work as an unemployed jobseeker. This would have been paired with regulations that oblige employers to offer open positions to recent dismissals, which could have resulted in Leena having to *‘drag on until Autumn anyway. And I did not want to do that anymore’.* Leena reports that she had not considered leaving work altogether until the negotiations came up, which is when she reports thinking, *‘Oh well, this is it.’*

Leena also reports taking time *‘just for myself.’* When asked, she elaborates having just started to *‘feel out this freedom’* and reports spending time on just herself *‘even though I have grandchildren.’* This type of accounting points to her awareness of the strong cultural norms concerning grandparents providing care for their grandchildren (Neuberger & Haberkern, 2014).

By her second interview, Leena has officially retired a couple of months earlier and is generally feeling good about her life, despite the smaller income. She also mentions that her husband is on a long sick leave and has applied for disability pension.

Leena also explains that she took the voluntary exit with financial package as a *‘soft landing’* to liberate herself from work as retirement age was so close that no abundance of work prospects existed for her.

- A: Yeah, it was so close for me, the retirement age, that I hadn’t even thought of applying for a job or not applying [laughs]. So it was pretty much wrapped up and ready for me because I don’t think I would’ve had any takers in work life anymore. Like, I had a [30 + -year] career in Posti, so there’s not much else I’d know how to do.
- Q: And that’s the thing, and also in your case, the thing that is of interest to this research project is precisely the transition from there, quitting work life, and moving on to retirement. So that’s one type of transition that occurs in people’s lives.
- A: Yeah. And this ended up being a sort of soft landing since (-) [laughs] I ended up retiring with the [financial adjustment package], leaving in advance, so I was liberated from work.
- (F34 Leena, Interview 2)

In her third and final interview, when asked about whether or not life has been as she expected it to be on retirement, Leena starts by answering in the affirmative but soon adds that her husband’s sick leave has been somewhat unexpected and prevented her from fully enjoying retirement as she had intended, meaning *‘That I would have been allowed to be my own master, for a while.’*

Leena has been somewhat roped into assisting with the electronic paperwork related to her husband’s situation, and this is something extraneous that *‘I really did not need at this stage’*, though she quickly adds that she is happy to help him, “of course.” Leena adds that, overall, she is happy with her long career and now her freedom, *“not having to go there [the workplace] anymore”.* She adds:

…if the employer had had a different stance towards employees, I might have actually kept on [working], why not. But the work wasn’t what it used to be when I started working.
(Leena F34, Interview 3)

Lacking employee appreciation was a common theme also among other participants either as a lack of small ways of showing appreciation (e.g., gifts) during the career, or at the end of it (Hannele F20), or related to the circumstances of the layoff (Ulla F36):

- … And the thing is that the employee has been completely forgotten. No appreciation, none whatsoever. We don’t even have enough appreciation from [upper management] to receive ten Christmas stamps. We just get a pretty letter, 'thanks for the year' and such. To top it all, they even took away our gifts for years of service. When you’ve been in the [organization] for 40 years you get a diploma, signed by the CEO. Well, apparently recently [laughs] some people sent them back and so they [the diplomas] were canceled and the gifts for years or service brought back.
- I: Right, so there was some upset over these diplomas?
- P: Yeah. I mean what am I going to do with a piece of paper like that?
- (Hannele F20, Interview 1)
- I: How would you describe those feelings?
- P: Well, it’s… Disgust. Or not disgust but what is it? Anger. It’s a feeling of anger alright. And we didn’t, the feeling of appreciation, like were we not appreciated or had we not done our jobs well since we always did well. The bottom line always showed that things were going well. Occasionally even a feeling of joy that I get to go free and then this horrible feeling of indifference towards the work and colleagues and the customers and everything. That was also a pretty strong one for me. A sort of, this sort of shrug. Oh well. I wasn’t (…) anything.
- (Ulla F36, Interview 1)

Like Hannele and Ulla above, Leena (F34) confirms to the interviewer that towards the end of her career, her work motivation declined, and the work became psychologically straining. A common experience, also articulated elsewhere in our data, was that the final 1−10 years at work represented a tumultuous time at Posti, marked by organizational changes that created either uncertainty or frustration:

- I: And apparently, things weren’t so hectic back then [at the start of your career] as they are [nowadays]?
- P: No, they weren’t, it was like when the work was done [for the day] you hang up your coat and go home. Now, this came about only in the last 10 years, that we should have [set] work hours (…) and it went to pieces from there. The problem was that [the amount of] mail is different each day, of course it’s not the same. It’s not a conveyor belt. Where you might be done for today by noon, tomorrow it could be at 3 pm, basically.
- I: So, it totally depends on the day?
- P: It totally depends on the day, yes. This is insane, that your workday is 7 hours [and] 39 minutes [long] and then (…) with the free time [breaks] factored in, that’s not the way it goes.
- (Ilmari M6, Interview 1)

Leena ultimately describes her retirement/home situation as something of a *‘crisis’* after having described it earlier in the interview in milder terms. She would have preferred to *‘settle down into this retirement life’* and make her own plans, whereas now she feels *‘sometimes like I am a bit programmed [laughs]. Like I cannot do [things] after all.’* To “*get away,’”“but also of course for my own fitness,”* she goes on walks and takes exercise classes.

### Case C, M11, Petri: requested dismissal followed by new meanings in life

#### Narrative summary

Petri’s case is an example of someone who asks to be dismissed to make room for others and begins increasingly focusing on homelife.

Petri mainly demonstrates narrative agency in the data by carefully steering away from generalizations and adding caveats to his more general situational assessments. There appears to be relatively little about the end of work and the beginning of retirement that, for him, requires the type of narrative sense-making that is present in the retirement accounts of involuntary dismissals (F21 Kaisa) or cases where expectations about life in retirement were not met (F34 Leena). It is possible that having a choice in the outcome of one’s collective negotiations near retirement (see also Kosonen et al., 2020) or in one’s daily routine after retirement plays a role.

Like Petri, other participants in the data also narrate their decision as involving a sacrifice of sorts to preserve someone else’s job, although in his case it appears to have been a fairly easy one. Petri explicitly frames his thinking on the topic as sense-making. This could be a manifestation of institutional pressure and narrative sense-making, which ultimately resulted from having limited options. Making sense-making explicit, Meretoja et al., (2022, p. 392) posit, could help people constructively deal with dominant cultural narratives.

The “lack of a clear vision” of the future, which Petri cites as an important factor in his willingness to leave work life behind, clearly resonates with the idea that narrative sense-making enables people to navigate the future. The vision was unclear, and he could not reasonably see himself partaking in it.

**Table V.** Interviews, M11 Petri.

1^st^ five months after work ended.
2^nd^ a little over four months since the 1^st^, nine months and two weeks since he quit working.
3^rd^ nearly four months since the 2^nd^ interview, 13 months since work ended.
4^th^ five months after the 3^rd^ interview, 18 months since work ended.
5^th^ a little over five months after the 4^th^ interview, nearly two years since work ended, a little over six months remaining until retirement.
6^th^ a little under seven months after the 5^th^ interview, a little over two years and six months since work ended, two weeks on retirement.

In his first interview, Petri articulates his skepticism towards what he perceives to be “lip-service” paid to the idea that ageing workers are or should be seen as a resource to their employers, stating that few companies actually think this way. He also speaks of physical limitations to the goals of extending working lives in a way that equates chronological aging with physical decline, although with the caveat that there may be some exceptions to this.

- A: I don’t know about extending work careers either because it is so few people who actually have the energy to actually extend them even if they were willing to do so. It’s just the case that there are many professions where, once you get to 63 and so on, [it becomes more difficult to pull through there]. Sure, a certain type of office job might still be manageable, but there are many professions in Finland where it is not possible at that age, except for a few individuals.
- (M11 Petri, Interview 1)

This was not an uncommon stance in the data and points to a popular notion of aging as decline (see Laceulle, 2018). Petri also mentions how the last five years of his career in Posti were marked by tensions related to the recurring collective negotiations that took place there in the 2010s.

At the time of the interviews, extending the statutory retirement age was a prominent issue in Finnish policy discussions, which is also reflected in the questions asked in the interviews and the responses to them. Below are examples from the broader data set that echo sentiments presented by Petri:

Of course it depends on the job but since I can only speak of shift work, I’ll say that if they raise the retirement age to something like 65 or 67, no one is going to retire alive from that. This is dead certain. You’ll be so worn out, so worn out, and then if we think about the retirement age going up and the [number of workers] going down, you’ll need to keep working more and more, like crazy. And these days a disability pension, it’s not so easy to get that.
(Ismo M9, Interview 1)
(…) the differences between professions haven’t been taken into account at all. For instance, if someone works in construction, for 40 years, then that person’s not going anywhere after that. It’s a good thing if they get out alive, or if they weld or paint the side of a ship for 40 years, they’ll be a total wreck.
(Juho M5, Interview 1)

Petri also explains that for him, a 40-year-long career was long enough, especially with the work having become “unclear” in recent years:

(…) and that’s why I left, like that this is enough, and what with this sort of general mess. The work was no longer, in a way, clear. You didn’t really know what was about to happen so I thought that this is enough, since I’ve been at it for 40 years now.
(M11 Petri, Interview 1)

In his second interview, Petri reports still enjoying life in retirement and builds on themes already present in the first interview, adding that one of the attractions of leaving work was being able to “take a breather and not following the clock or schedules” and “to just live” and that these sentiments have not changed since retiring. Petri re-iterates that he did have a 40-year career in Posti and looking at the company’s present-day situation, he has no desire to return. A 40-year-long career appears here as a milestone that he had just managed to reach.

But if we’re just talking about free time and such, the thing is, I did work for a pretty long time, 40 years of it, I did get the full 40 years since I left at the start of the year and I think it was [specific date a few months later] that I was still on their books, so it did get to the full 40. So in that sense I have no great longing [to return there]. And if we’re being honest about the present-day situation in Posti, I think it’s pretty dire. (Petri M11, Interview 2)

Overall, Petri’s second interview is largely built on logical sense-making and cause-effect: *since I hit the 40 years and it was pretty dire, I decided to quit*. It is possible that a voluntary work exit could be less of a rupture in the expected trajectory of a working person’s life and thus may require less narrative sense-making that manifests narrative agency.

In his third interview, Petri no longer explicitly reminisces about Posti and other matters appear to have gained a larger role in his life, and there is very little narratively agentic content. Work life was not mentioned until Petri began discussing his pension accrual.

In his fourth interview, Petri confirms that money has never really been *‘the point’* for him during his career, and that family matters were always far more important. When asked about his upcoming pension age, Petri states that he is somewhat unclear on the exact age when he can apply for it and that he is not sure whether thinking about *‘retirement stuff is such a good thing at all.’* He has found that his pension will not be *‘all that great,’* maybe just better than what he is currently getting from his labor union during unemployment, *‘although I was a manager and all, and for quite a long time too,’* implying an unfulfilled expectation that his pension should have been better for the work that he did and the length of his career. There is a sense of avoidance in Petri’s account that could speak of structural bounds and one possible response to them being nonaction.

Throughout his fifth interview, Petri is careful not to make too many generalizations. He keeps adding what could be caveats to statements that would otherwise paint younger workers as categorically less motivated, all levels of management above himself as incompetent and out-of-touch with daily realities of postal work, or Posti as singular in their organizational restructuring in 2000-2010s Finland. These statements can be seen as examples of *narrative awareness*, an aspect of *narrative agency* (Meretoja et al., 2022).

- A: Yeah, exactly then. The last 10 years of my career. I am sure, increasingly, year by year, it always became harder in a way, the savings targets, which [were a prominent feature of] any corporation in Finland, so that was let’s say from the end of the 2000s pretty much until now, the 2010s, and it was a whole lot of saving up and cooperative negotiations. And then I started getting those duties in the 2010s, the negotiations and running those [duties]. In a way that was a lot of work, and it is not pleasant work, by any means. There was another round of negotiations at some point. Going over those things with people, it was probably one of those things that pretty much directed my thinking that this was not the most pleasant job.
- (Petri M11, Interview 5)

Petri confirms that workplace atmosphere and a lack of clarity were factors in his eagerness to leave work when he did, adding that this had been the state of affairs for *‘many years already,’* adding further: *‘and it doesn’t seem like things have gotten better. ’* For Petri, leaving work was a matter of making it happen and his own choice.

Petri reports that the most important reason for his loss of work motivation was the *‘lack of a clear vision of the future,’* and that the cut-back instructions handed down from higher management were not always congruent with the realities of the actual work, whereas he himself was involved in the work and saw *‘how stuff gets done.’* He also notes that exchanging experienced workers with *‘younger ones’* is not so simple.

To Petri’s mind, there were *‘too many cooks’* in the proverbial kitchen *‘and in the end it amounted to absolutely nothing.’* This period of change, Petri estimates, took up the last 10 years of his career, with things *“getting harder each year.”* Part of this appears to have been the regular collective negotiations that he reports had been undertaken since 2010, the handling of which was a part of his job, *‘not a nice job at all,’* an unpleasant duty that potentially reflected on his job overall.

In his sixth and final interview, when asked to envision what the future might hold for him, Petri quickly turns the discussion to his loved ones and their life situations, and how he wishes that his wife and children have things going well for them. He also states that work, for him, for the most part, is in the past. Envisioning a future for oneself, in ways other than work, does not involve any grand schemes, as is true for several participants.

Petri confirms that he is not under any stress to go back to work or apply for a job, stating that *‘it would have to be a pretty (…) extraordinarily interesting job,’* but quickly adding that he has not completely closed the door on that possibility. He is not actively looking for a job but might be open to being offered smaller jobs. In the meantime, he plans to *‘just be, as they say, and do these daily routines that I have.’* He has found that he has no trouble finding things to fill up his days and is surprised by how much time activities that he did not have the time or energy for while still working could take up, such as exercise and reading.

## Discussion

The present study concurs with Meretoja et al. (2022) in that the stories people tell themselves and each other can delineate what they see as possible. The participants exhibited a limited “sense of the possible” that could signal a need for proactive means to help older employees (or indeed employees of all ages) gain awareness of and potentially resist the stories they live by (see Meretoja et al., 2022).

The data included both white-collar and manual laborers, staff, and managers. Long careers of 30−40 + years are evoked as a narrative milestone when justifying retirement-timing, as having already done 'enough' (see Petri M11 and Leena F34) or not knowing where else to work anymore (see Kaisa F21).

Mentions of long work careers also appear in the rest of the 20-person data set’s interviews, either as a personal achievement or a sufficient length for a person’s work career. This quantification of the career length indicates the presence of a broader norm, either on the organizational or societal level. In the three cases under closer analytic scrutiny here, the primary motivation for leaving work was, somewhat surprisingly, not to preserve their physical health. Instead, reference was made elsewhere in the data to occupational differences that make working longer an impossibility to many due to physical demands on the job (see Ismo M9 and Juho M5).

Having no desire to go back to work often co-occurred with a lack of clarity related to the job itself in recent years (see Petri M11) or a lack of employee appreciation (see Leena F34, Hannele F20, and Ulla F36). Some of the 20 participants reported that Posti used to have a more flexible work culture before the 2000s, when worktime came to be more regulated (see Ilmari M6); at the same time, increasing work demands were sometimes used as rationale for recommended work exits (see Mika M2, in Table II).

Allen et al. (2021) have observed that flexible work arrangements (FWAs) may actually have some utility against ageist “metastereotypes” regarding ageing at work: their findings showed that *'greater access to FWAs was associated with lower perceptions of negative stereotypes in the workplace, and both greater access to and use of FWAs weakened the negative association of perceived negative stereotypes with work engagement'* (Allen et al., 2021, p. 410). The addition of structural flexibility to extend work lives is supported by a longitudinal survey study (*N* = 1,466) conducted in the same research project on the same cohort of postal workers as the interview participants in this study (Neupane et al., 2022).

The narrative of older workers needs to change to one where they can envision themselves as continuing to work. To achieve this, more than words are needed, since the data also shows older workers’ distrust of platitudes and lip-service paid to the idea that they are an appreciated asset (see Petri M11). Change requires concrete and convincing action by employers and policymakers to support such claims (Hyde & Dingemans, 2017).

Our empirical analysis clearly confirms that all efforts to extend work lives need to take stock of aging concepts that are used to negotiate delayed or early retirement. Creating a more sustainable work culture entails examining potential counter-narratives to what Lakoff & Johnson, (1999) see as a metaphor underpinning collective storytelling in “Western” societies, i.e., the conceptualization of life (and, therefore, work life) as a survival battle. This is hardly an attractive image if the alternative is freedom brought about by retirement, as exemplified in Leena’s (F34) account about her unfulfilled retirement plans (see also Apouey, 2022; Grødem & Kitterød, 2022; Repetti & Calasanti, 2018).

In all, through a carefully designed three-year longitudinal follow-up, the three cases discussed here shed important light on retirement situations from different perspectives. Case A articulated the difference between one’s own and other people’s perceptions of unemployment, Case B grappled with idealized versions of retirement transforming into something else due to events not in one’s control, and Case C increasingly found meaning in family life and taking time with simple daily routines at home.

In terms of agency, the longitudinal analysis yielded some differences for each participant’s individual development over time: Where Kaisa (F21) came to question her sense of shame over her lay-off, Leena’s (F34) expectations of a period of freedom fell victim to a harsher reality of being “*programmed*” by another which she ultimately found ways to experience freedom from, to a more limited degree. Meanwhile, Petri (M11) adopted a more causal stance to his decision to quit where the structures of work life, first within and finally outside Posti, played a relatively large role.

Petri’s (M11) reported increase in happiness with life as chosen daily routines without strict schedules resonates with findings by Bauger and Bongaardt (2016) on *‘*a heightened sense of agency’ as a constituent of well-being in retirement, centering around a “freedom to”. Relationships with family members during retirement were at first peripherally present and came to be a more prominent theme through the follow-up for Leena (F34), who reported taking time for herself “*even though [she has] grandchildren”* and later ended up taking care of her husband’s paperwork for him and feeling *‘programmed’* because of it. While family can be an important source of meaning in retirement (Halama et al., 2021), it also appeared as a potential source of tension in Leena’s interviews. This latter point also resonates with Bauger and Bongaardt (2016) findings that retirees may be quite protective of the autonomy they have gained in retirement.

The participants demonstrated aspects of narrative agency, mainly narrative or perspective awareness. Two of the three cases demonstrated a 'sense of the possible' in the negative as an unfulfilled potential (case B, Leena F34), and in the positive as the ability to *‘roll over and read a book’* (case C, Petri M11). The participants were careful not to “dream big” in terms of future wishes or significant life changes. This could indicate a structurally limited 'sense of the possible'—or simply, satisfaction with life as a gentle daily routine outside of wage labor.

Case A, F21, Kaisa, grapples with the meaning of work, and her thinking regarding her own value as an unemployed person undergoes a shift during follow-up. Her case history resonates well with the findings of Bengtsson & Flisbäck, (2017, p. 62) regarding “how existential questions arise in the ‘forced’ retirement process”. Kaisa presents a practical example of 'explorative ethical inquiry' (Meretoja et al. 2022, p. 393), questioning narratives that she frames as dominant regarding life in retirement. This is proof of how narrative agency provides latitude and allows individuals in life transitions to also oppose culturally dominant model stories.

It is also worth noting that organizations that narrate their own future visions in an unclear manner could discourage skilled older workers from pursuing longer careers. Posti underwent a series of organizational changes over the 2010s, and based on the data, the way these changes were communicated and implemented appears to have left a bitter after-taste and potentially influenced older employees’ decisions not to continue working there. This is particularly visible in Petri’s (M11) case.

Perhaps in a bid to extend work lives sustainably, employers should be tasked with fostering employees’ ability to see workplaces and extended careers as what might be termed “spaces of the possible.” Aging at work with new flexibilities could in important ways even be analogous to life in retirement. Such a workplace would arguably enable workers to see future opportunities for themselves and make plans for a future at work (see also Ng & Law, 2014).

The paradigm shift from a “right to retire” to a “duty to work” (Gould & Saurama, 2003) appears in our data as a tension between the new and the old paradigm: older workers that started their careers in the 1970s and were retiring in the 2010s carried in their narratives the sense of a deserved retirement after 40 years, but were also faced with the idea that they might be called back to work during a period of unemployment preceding retirement. The shift in paradigm, for them, does not appear to be motivating. Conversely, their concern for the employment prospects of younger generations was often mentioned as a further motive to vacate the workplace, echoing the inter-generational contract of the early retirement paradigm, when a financial opportunity to leave work arose.

Similar shifts in retirement policies in the 2000s towards active ageing were found by Hofäcker et al. (2016) who compared data from the UK, Germany and Japan. Their findings on trends in voluntary/involuntary retirement over time offer some encouragement in terms of the possibility to extend work lives sustainably. Their findings on Japan, however, point to a potential upper limit of doing so, which echoes the analytic findings in this study:

*“Finally, trends over time suggest an improvement with regard to choice as the incidence of voluntary retirement increased in England and Germany. Yet in Japan, where actual retirement ages were high already in the 1990s, the shift to longer employment apparently comes at the cost of higher ill-health-related involuntary retirement. This suggests that there may be limits to the extension of working lives.”* (Hofäcker et al., 2016, pp. 60−61).

## Conclusion

The aim of this study was to offer insight into the broader question of postponing retirement age, and the work exit process by applying meticulous qualitative longitudinal research (QLR). Our aim was to see if a) Meretoja and colleagues’ (2022) model could be applied to analyzing interview transcripts and b) if this model could illuminate the agency of older workers and potential changes to it over time. Using QLR meant following or 'walking alongside our participants' and analyzing the ways they narrate their work exit and retirement processes in retrospect as well as the nuances of narrative sense-making and agency.

The analysis revealed a limited “sense of the possible’ (Meretoja et al., 2022) in retirement transitions. Such limitations are an indication of the structurally bound nature of the participants” unfolding life situations and their individual agency. While retirement may ideally be seen as a time of freedom, it is not freedom without limits or with endless possibilities. Rather, it is freedom to “roll over in the morning and read a book,” to not go back to an increasingly physically and/or mentally taxing job. It can also be described and justified as making way for younger colleagues. In fact, retirement decisions can be seen as analogous to individual agency more generally, bounded by and reproducing social structures (see Flaherty, 2012).

The findings indicate that retirement is a “space of the possible” in the popular imagination, and decisions regarding it are treated accordingly. One solution to potentially extending work-lives, then, could be to make work life more of a “space of the possible” by adding structural flexibility, e.g., to working times, hours, and conditions, and providing support measures to employees of all ages, when necessary. Extending work lives sustainably requires employers and policymakers to agree that older workers are a key asset to retain in the workplace (Hyde & Dingemans, 2017). This necessitates adjustments and negotiated flexibility, which signal to older workers that they truly are valued.

Ascribing to the idea that narratives allow people to navigate their futures also commands further study on the clarity of communicating organizational changes and doing this in a way that fosters a “sense of the possible” (Meretoja et al., 2022) at work. This would undoubtedly prove beneficial and help to retain skilled and motivated older workers.

## Data Availability

Because the dataset is very large, recorded, and transcribed only in Finnish, and has not been anonymized or translated into English in its entirety, it has yet to be made available. However, the data will be made available on reasonable request.
